# Antinociceptive and Anti-inflammatory Effects of *Pistacia vera* LeafExtract in Mice

**Published:** 2011

**Authors:** Hossein Hosseinzadeh, Effat Behravan, Mohammad M Soleimani

**Affiliations:** a *Pharmaceutical Research Center, Department of Pharmacodynamy and Toxicology, School of Pharmacy, Mashhad University of Medical Sciences, 91775-1365, Mashhad, Iran.*; b *Department of Pharmacodynamy and Toxicology, School of Pharmacy, Mashhad University of Medical Sciences, Mashhad, Iran.*

**Keywords:** *Pistacia vera*, Antinociceptive, Anti-inflammatory, Hot plate test, Writhing test, Xylene-induced ear edema, Cotton pellet test.

## Abstract

*Pistacia vera *L., a member of Anacardiaceae family, has been used for sedation and analgesia in traditional medicine. In this study, the antinociceptive and anti-inflammatory effects as well as acute toxicity of the aqueous and ethanolic extracts of *P. vera *leaves were investigated in mice. The antinociceptive activity was studied using hot plate and writhing tests. The effect of the extracts against acute inflammation was determined using xylene-induced ear edema and the activity of the extracts, against chronic inflammation, was assessed using the cotton pellet test. The LD_50 _values of the infusion and maceration extracts were 0.8 g/Kg and 0.79 g/Kg, respectively. The aqueous and ethanolic maceration extracts of the *P. vera *leaves at the doses of 0.4 g/Kg and 0.5 g/Kg (IP), respectively, showed antinociceptive effects. The pretreatment of naloxone (2 mg/Kg, SC) inhibited the activities of extracts in hot plate test, but naloxone at the same dose could not inhibit the antinociceptive activity in writhing test. The extracts also showed anti-inflammatory effects in acute and chronic anti-inflammatory tests. The ethanolic extract was as effective as diclofenac in both inflammatory tests. The aqueous and ethanolic extracts of *P. vera *leaves demonstrated central and peripheral antinociceptive activities dose-dependently and the central effect may be mediated by opioid system. The extracts also demonstrated anti-inflammatory effects against acute and chronic inflammation.

## Introduction

The chronic inflammatory diseases are one of the major health problems worldwide and non-steroid anti-inflammatory drugs (NSAIDs) are the most prescribed drugs for the treatment of inflammatory diseases. Although the NSAIDs provide the patients with symptomatic relief, they do not modify the pathogenesis of inflammation and do not reduce the disabling bone and cartilage damage (1). Therefore, there has been substantial amount of research for finding pain relief medications and reducing the inflammation. The antinociceptive and anti-inflammatory effects of some plants such as *Zataria multiflora *(2, 3), *Crocus sativus *(4) *Zhumeria majdae *(5) and *Elaeagnus angustifolia *(6) *Urtica pilulifera *(7) *Rosa damascena *(8) have been reported previously, and the results proved good analgesic and anti-inflammatory activities.

*Pistacia vera *L., a member of Anacardiaceae family, has sedative and tonic effects (9). In folk medicine, it has been used as analgesic, carminative, astringent, stomachic, aphrodisiac, antitussive, diuretic, and expectorant (10). The kernels of *P. vera *are remarkably rich in linoleic and linolenic acids, which are the vital fatty acids for human health (11). In modern pharmacology, Pistacia species have been reported to have various biological effects such as antiatherogenic, hypoglycemic, antioxidant, antiprotozoal, analgesic and anti-inflammatory (12-13). The aqueous extract of leaves and nuts of *P. vera *has antiemetic activity in young chickens with peripheral and central mechanisms (14). In a study, *P. vera *gum exhibited neuroprotective effects against ischemia (14). In different studies, *P. weinmannifolia *showed a potent antioxidant activity (16-17). The ethanolic extract of *P. vera *gum has a protective activity against liver damage induced by CCL4 in rats (16), and in another study, ethanolic extract of *P. vera *gum showed antinociceptive effect (19).

The chemical constituents of the Pistacia genus were studied, and monoterpenes (18), tetracyclic triterpenoids (21), flavonoids (22), and other phenolics including gallic acid (16-23) and essential oils (24) were found. Six gallotannins and seven flavonoid glycosides were isolated from *P. weinmannifolia *(25). *P. terebinthus *has acute and chronic anti-inflammatory effects, and its main ingredient, oleanonic acid which is a 3-Oxotriterpenoid, has leukotriene anti-synthetic property (12). In a study, *P. anacardiaceae *showed a noticeable analgesic and anti-inflammatory effect at *in-vivo *tests (26). Since I.R. Iran is one of the main origins of pistachio and among the species of Pistacia genus, *P. vera *is the most economically important one, we investigate the anti-inflammatory and analgesic effect of this species.

## Experimental

*Plant material*

The plant was collected from south of Khorasan (Gonabad, Iran), dried in shadow, and ground. The voucher samples were preserved for reference in the Herbarium of Pharmacognosy Department, School of Pharmacy, Mashhad University of Medical Sciences, Mashhad, Iran (with Voucher No. of 165-2502-2). The plant powder was extracted using aqueous infusion and maceration with ethanol. For the aqueous infusion, hot water was added to plant material (100 g) and boiled for 15 min, then filtered through a clean cloth. For ethanolic extraction, the plant powder was macerated in ethanol (80%, v/v) for 72 h and the mixture was subsequently filtered (5, 27).

*Animals*

Male and female albino mice of 25-30 g were used. Animals were obtained from Razi Institute and Pharmacology Lab in Mashhad University of Medical Science and were housed under a 12/12 h light/dark cycle at 21 +/- 2°C with free access to water and food. The handling and use of animals were in accordance to the institutional guidelines and all experiments were carried out in accordance with current and ethical guidelines for the care and use of laboratory animals.

*Acute toxicity tests*

Different doses of extracts were injected into groups of six mice intraperitoneally (IP) and the number of deaths was calculated at 48 h after treatment. LD_50 _values and corresponding confidence limits were determined using the Litchfield-Wilcoxon method (PHARM/PCS Version 4)**.**

*Antinociceptive study*

*Hot plate test*

*Evaluation of antinociceptive activities of aqueous and ethanolic extracts*

According to the results of acute toxicity test, the highest doses without mortality for aqueous and ethanolic extracts were 0.4 g/Kg and 0.5 g/Kg respectively. We chose 4 groups of doses based on 100, 70, 40 and 10% of the highest dose for aqueous (0.04, 0.16, 0.28, 0.4 g/Kg) and ethanolic extracts (0.05, 0.2, 0.35, 0.5 g/Kg). We used saline and morphine (10 mg/Kg IP) as negative and positive controls.

In hot plate test, the aqueous and ethanolic extracts were evaluated in six groups of 6 mice. The temperature of the metal surface was 55°C and the latency to a discomfort reaction (jumping or licking paws) was measured. The cut-off time was 40 sec (28). The responses were recorded in 0, 30, 60, 90 and 120 min after administration.

*Effect of naloxone on antinociceptive activity of aqueous and ethanolic extracts*

According to the previous test and its results, we chose the doses of 0.4 g/Kg for aqueous and 0.35, 0.5 g/Kg for ethanolic extracts. Two separate tests were performed (6 groups for aqueous and 8 groups for ethanolic extracts). Pretreatment with Naloxone (2 mg/Kg SC) was carried out 15 min before the administration of a morphine group (10 mg/Kg IP) and one group for each extract. We also used each extract separately and normal saline (10 mg/Kg) and morphine (10 mg/Kg) as negative and positive controls.

*Writhing test*

The aqueous and ethanolic extracts were administered to the groups of 6 mice in two doses, and 30 min later an intraperitoneal injection of 0.7% v/v acetic acid solution were given to mice (0.1 mL/10 g). The number of writhes was counted for 30 min (29).

*Anti-inflammatory study*

*Xylene-induced ear edema*

Mice were divided into 5 groups of six. Sixty min after the IP injection of the extracts or diclofenac (as positive control) or normal saline (as negative control), one drop of xylene was applied to the anterior surfaces of the left ear and the right ear was considered as a control. Two h after the Xylene application, animals were killed and both ears were removed. Circular sections were applied (4.5 mm) and weighed. The difference of weight in the left ear section from that of the right was considered as edema (30).

*Cotton pellet granuloma*

The chronic anti-inflammatory activity was tested using granuloma pouch method**. **We used 4 groups of mice: 2 groups for extracts, one normal saline as negative and diclofenac as positive controls. Sterile cotton gauze dental packs were washed with a solution of Cetrimide-C. After total anesthesia, dorsal shoulders of mice were shaved and disinfected. Two cotton pellets impregnated with 0.4 mL of an aqueous solution of ampicillin were implanted in shoulders of mice (SC), one on each side. In the following seven days, the animals were treated with extracts or diclofenac. On day 8, the mice were killed and the pellets and surrounding granulation tissues were dried at 60°C for 24 h. The weight of granuloma and the percentage inhibition of inflammation were measured for all the groups (30).

*Statistical analysis*

Statistical analysis was carried out using one-way ANOVA. Level of p < 0.05 was considered as significant. The data were reported as mean ± SEM and tested with analysis of variance followed by the multiple comparison test of Tukey-Kramer. 

## Results and Discussion

The LD_50_ values of the infusion and maceration extracts were 0.8 g/Kg and 0.79 g/Kg, respectively and the maximum non-fatal doses were 0.4 g/Kg and 0.5 g/Kg respectively.

**Figure 1:**
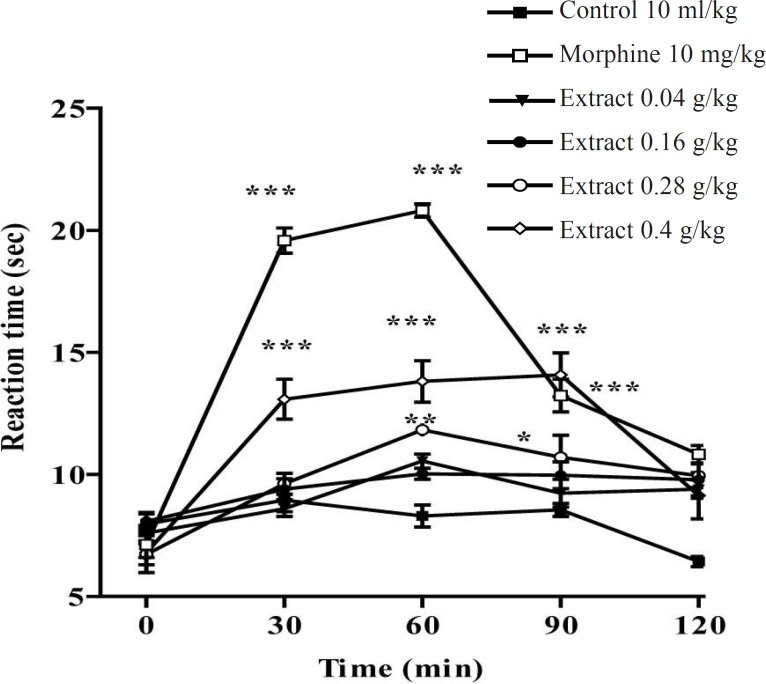
Effect of the aqueous extract of *Pistacia vera *leaf and morphine (IP) on the pain threshold of mice in the hot plate test. Each point represents the mean ± SEM of reaction time for n = 6 experiments on mice, compared with control. *: p < 0.05; **: p < 0.01; ***: p < 0.001; Tukey-Kramer test

**Figure 2:**
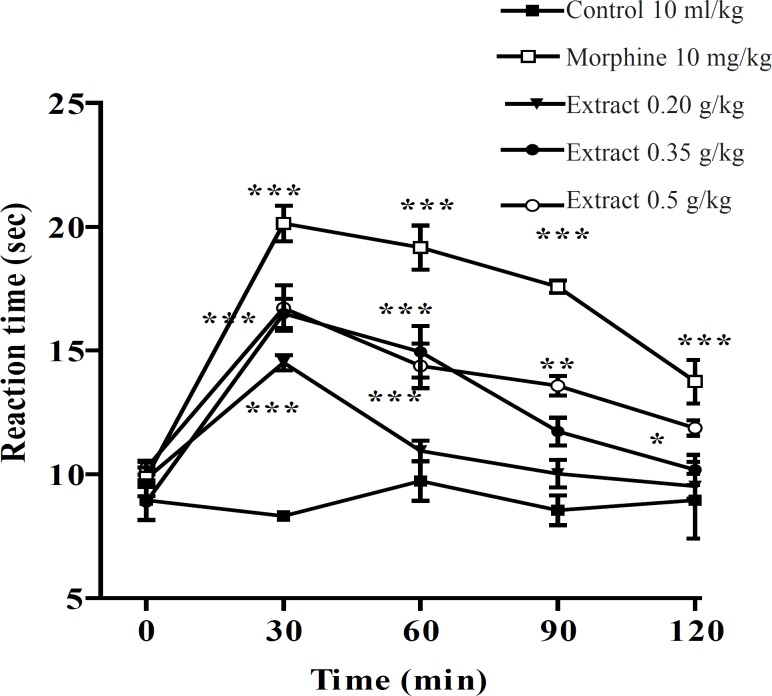
Effect of the ethanolic extract of *Pistacia vera *leaf extract and morphine (IP) on the pain threshold of mice in the hot plate test. Each point represents the mean ± SEM. of reaction time for n = 6 experiments on mice, compared with control; *: p < 0.05; **: p < 0.01; ***: p < 0.001; Tukey-Kramer test

In the hot plate test, the administration of the aqueous and ethanolic extract demonstrated a dose-dependent antinociceptive activity after 30-60 min of treatment (Figures 1 and 2).

**Figure 3:**
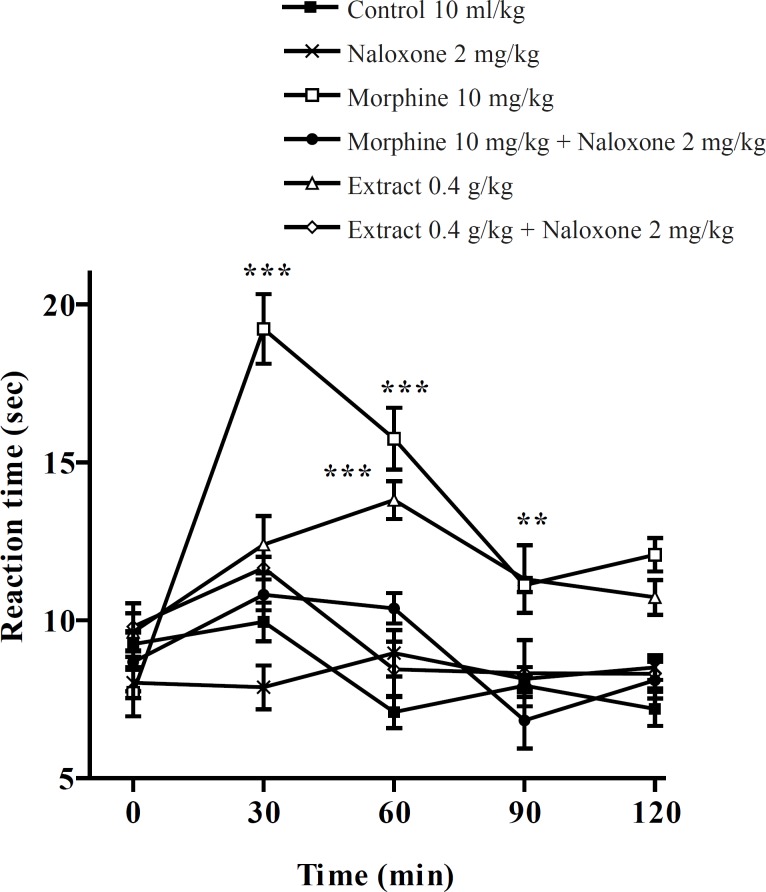
Effect of naloxone on the aqueous extract of *Pistacia vera *leaf and morphine (IP) anti-nociceptive activity in mice (hot plate test). Each point represents the mean ± SEM of reaction time for n = 6 experiments on mice, compared with control, **: p < 0.01; ***: p < 0.001; Tukey-Kramer test

**Figure 4:**
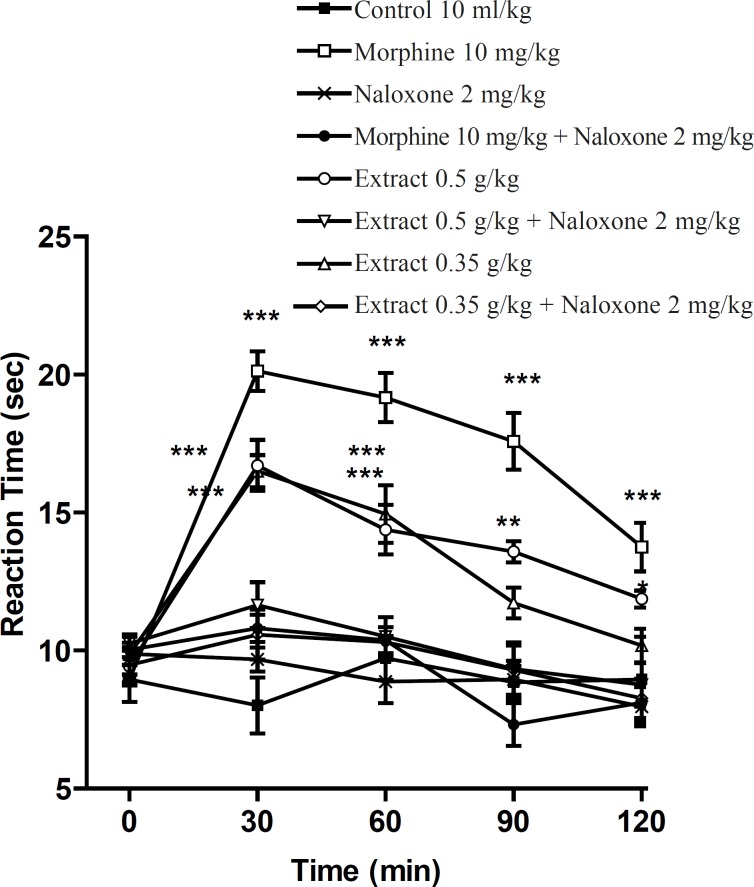
Effect of naloxone (SC) on the ethanolic extract of *Pistacia vera *leaf and morphine antinociceptive activity in mice (hot plate test). Each point represents the mean ± SEM of reaction time for n = 6 experiments on mice, compared with control, *: p < 0.05; **: p < 0.01; ***: p < 0.001; Tukey-Kramer test

Pretreatment with naloxone (2 mg/Kg, IP) inhibited the antinociceptive activity of the aqueous extract (0.4 g/Kg, IP) and morphine (10 mg/Kg, IP), sixty min after the injection. Only at the time of 60 min, extract (0.4 g/Kg) was more effective than normal saline and the antinociceptive effect showed a plateau effect between 30 to 90 min. The ethanolic extract also showed the dose-dependent antinociceptive activity that was inhibited by naloxone (Figures 3 and 4).

**Figure 5:**
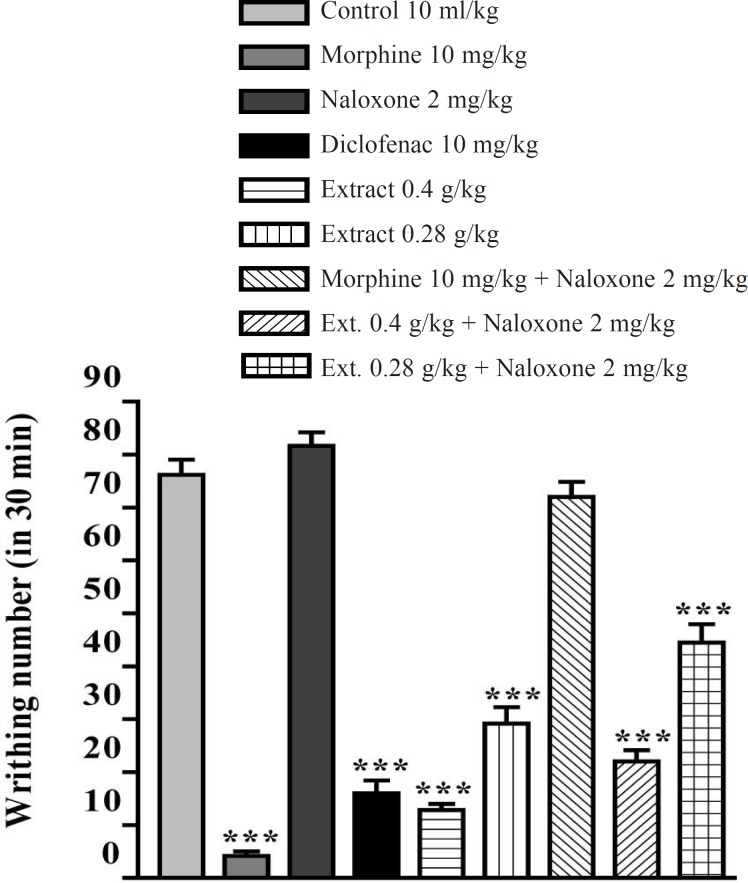
Effect of naloxone (SC) on antinociceptive effect of the aqueous extract of *P. vera *leaf on acetic acid-induced writhing test in mice. The values are shown as mean ± SEM for n = 6, compared with control, ***: p < 0.001; Tukey-Kramer test

**Figure 6:**
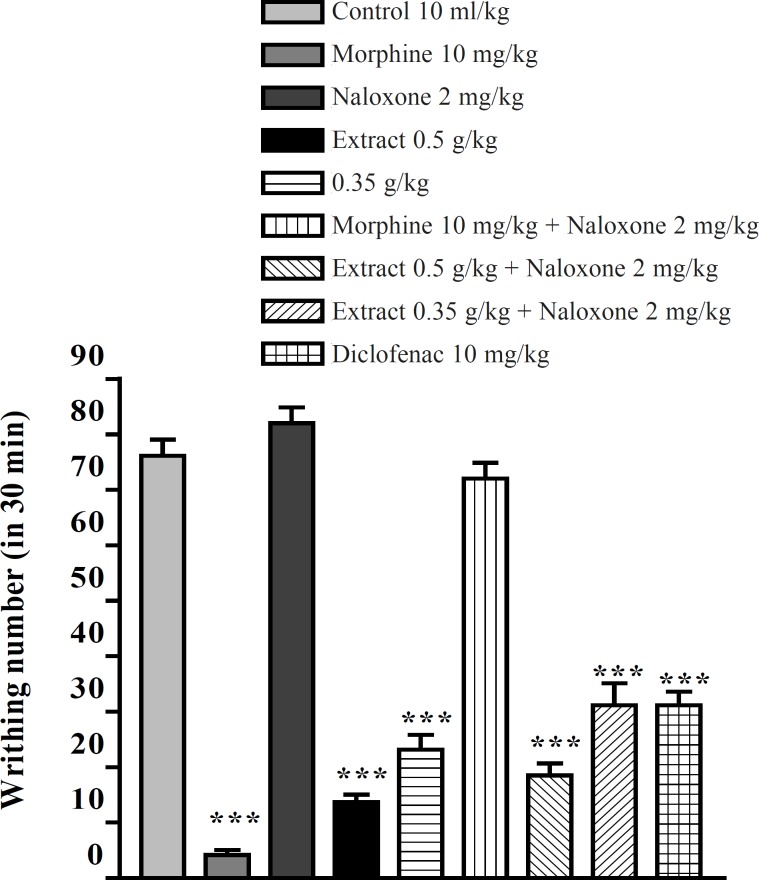
Effect of naloxone (SC) on antinociceptive effect of the ethanolic extract of *P. vera *leaf on acetic acid-induced writhing test in mice. Values are shown as mean ± SEM for n = 6, compared with control, ***: p < 0.001; Tukey-Kramer test

The aqueous and ethanolic extracts of *P. vera *leaves significantly reduced the number of mouse abdominal constrictions induced by an acetic acid solution. There was no significant difference between diclofenac (10 mg/Kg), morphine (10 mg/Kg) and the extracts in reduction of writhing numbers. Pretreatment with naloxone (2 mg/Kg IP) after the IP injection of extracts could not inhibit the antinociceptive activity of both extracts (Figures 5 and 6). In the xylene-induced ear edema study, the highest doses of aqueous and ethanolic extracts showed significant anti-inflammatory activities with %56 and %66 of inflammation and inhibition after 1 h, respectively. The ethanolic extract showed higher activity against the acute inflammation than did the aqueous extract (Tables 1 and 2).

**Table 1:** Effect of the intraperitoneal doses of *Pistacia vera *aqueous leaf extracts on Xylene-induced ear swelling in mice

**Treatment**	**Dose**	**Ear swelling (mg)**	**Inhibition (%)**
**Normal saline**	10 mg/Kg	3.8 ± 0.3	-
**Diclofenac**	15 mg/Kg	1.3 ± 0.3***	66.31
**Aqueous extract**	0.4 g/Kg	1.6 ± 0.3 **	56.76
**Aqueous extract**	0.28 g/Kg	2.8 ± 0.3*	44.74
**Aqueous extract**	0.16 g/Kg	2.8 ± 0.4	26.25

Values are shown as mean ± SEM for n = 6 mice, ***: p < 0.001; **: p < 0.01; compared with control (Tukey-Kramer).

**Table 2:** Effect of the intraperitoneal doses of Pistacia vera ethanolic leaf extracts on Xylene-induced ear swelling in mice

**Treatment**	**Dose**	**Ear swelling (mg)**	**Inhibition (%)**
**Normal saline**	10 mg/Kg	4.6 ± 0.4	-
**Diclofenac**	15 mg/Kg	2.0 ± 0.4*	55.86
**Ethanolic Extract**	0.5 g/Kg	1.5 ± 0.3 **	66.95
**Ethanolic Extract**	0.35 g/Kg	4 ± 0.7	12.47

Values are shown as mean ± SEM, n = 6, *: p < 0.05; **: p < 0.01, compared with control (Tukey-Kramer)

In the chronic inflammation (cotton-plate) test, both extracts showed significant and dose-dependent anti-inflammatory activity. A dose of 0.5 g/Kg of ethanolic extract, showed a higher effect than diclofenac 15 mg/Kg (Tables 3 and 4).

**Table 3:** Effect of the intraperitoneal doses of *Pistacia vera *aqueous leaf extracts (consecutive for 7 days) on the weight of granuloma in mice

**Treatment**	**Dose**	**Cotton pellet (mg)**	**Inhibition (%)**
**Normal saline**	10 mL/Kg	11.0 ± 0.8	-
**Diclofenac**	15 mg/Kg	4.0 ± 0.3***	64.12
**Aqueous Extract**	0.4 g/Kg	4.3 ± 0.5***	61.11
**Aqueous Extract**	0.28 g/Kg	5.1 ± 0.5***	53.31

Values are shown as mean ± SEM, n = 10, ***: p < 0.001 compared with control (Tukey-Kramer)

**Table 4:** Effect of the intraperitoneal doses of *Pistacia vera *ethanolic leaf extracts (for 7 consecutive days) on the weight of granuloma in mice

**Treatment**	**Dose**	**Cotton pellet (mg)**	**Inhibition (%)**
**Normal saline**	10 mL/Kg	11.0 ± 0.8	-
**Diclofenac**	15 mg/Kg	4.0 ± 0.3***	64.12
**Ethanolic Extract**	0.5 g/Kg	3.5 ± 0.5***	68.48
**Ethanolic Extract**	0.35 g/Kg	5.5 ± 0.7***	50.04

Values are shown as mean ± SEM, n = 10, ***: p < 0.001 compared with control (Tukey-Kramer)

The present results showed that the aqueous and ethanolic extracts of *P. vera *leave have peripheral and central antinociceptive activity. The extract also demonstrated anti-inflammatory effects against acute and chronic inflammation.

The ethanolic extract showed more analgesic activity than aqueous type. Different studies showed that most of the ingredients of Pistacia species are oil-soluble. Ansari shows 3 triterpenes as major components of *P. integerrima *(31)*. *One of the most identified active triterpenoids is oleanolic acid, which was acquired from *P. *terebinthus (10). In addition, oleoresin was shown to possess a marked anti-inflammatory activity (26). *α*-Pinene, a monoterpene-type of compound, is the dominant component in the essential oils obtained from leaves of *P. vera *(29.2%) and resin of *P. lentiscus *(21.7%) (32). *α*-Pinene showed anti-inflammatory effect (31). We can conclude that these oily-soluble components are the important cause of antinociceptive and anti-inflammatory effects of *P. vera *leaves.

With respect to the LD_50_ value and in comparison with a toxicity classification (34), the extracts were semi-toxic.

The aqueous and ethanolic extracts showed antinociceptive activity in the hot plate test and this effect was inhibited by naloxone. The hot plate test is a specific central antinociceptive test (35). Thus, it is suggested that the antinociceptive activity part of *P. vera *leaf may be mediated through a morphine-like and central activity.

As the preliminary phytochemical results indicated, the antinociceptive and anti-inflammatory effects of extracts may be due to their content of triterpenoid tetracycline. Other studies have demonstrated that triterpenes have significant antinociceptive and anti-inflammatory activities (31).

The antinociceptive activity of opioid agonist and non-steroidal anti-inflammatory agents can be determined using the writhing test (36). The antinociceptive activity of the extracts was not inhibited by naloxone completely, so other mechanisms of action, such as cyclooxygenase inhibition and peripheral mechanism may be considered.

In Xylene-induced ear edema test, increasing the vascular permeability caused edema in tissue. Following the stimulation by Xylene, mediators are released and induce the dilation of arterioles and venules. We can suggest that *P. vera *may have inhibitory effects on the release of mediators and also has a membrane-stabilizing effect that reduces capillary permeability.

The extract also reduced cotton pellet-induced granuloma, thereby suggesting its activity in the proliferative phase of the inflammation. It is concluded that extracts shows anti-inflammatory effect by inhibition of mediators› synthesis or other mechanisms like steroid factors.

A study on volatile essence of 3 Pistacia species showed that hydrocarbon monoterpenes were the main part. Oleanic acid is one of the best known biologically active triterpenoid (a 3-oxotriterpenoid), which has anti-inflammation and leukotriene inhibition synthesis effects (12).

It is concluded that the aqueous and ethanolic extracts of *P. vera *leaf have peripheral and central analgesic effects. Opioid receptors and the inhibition of cyclooxygenase enzyme may mediate these activities. The extracts also have activity against acute and especially chronic inflammation. The chemical constituents responsible for the pharmacological activities remain to be investigated.

## References

[1] Ford-Hutchinson AW, Bray MA, Cunningham FM, Davidson EM, Smith MJ (1981). Isomers of leukotriene B4 possess different biological potencies. Prostaglandins.

[2] Hosseinzadeh H, Ramezani M, Salmani G (2000). Antinociceptive, anti-inflammatory and acute toxicity effects of Zataria multiflora Boiss extracts in mice and rats. J. Ethnopharmacol.

[3] Owlia P, Pirveicy H, Saderi H, Rezvani MB, Mansouri (2004). Evaluation of the antimicrobial effects of extract of Zataria multiflora against oral Streptococci. Iranian J. Pharm. Res.

[4] Hosseinzadeh H, Younesi HM (2002). Antinociceptive and anti-inflammatory effects of Crocus sativus L stigma and petal extracts in mice. BMC Pharmacol.

[5] Hosseinzadeh H, Ramezani M, Fadishei M, Mahmoudi M (2002). Antinociceptive, anti-inflammatory and acute toxicity effects of Zhumeria majdae extracts in mice and rats. Phytomedicine.

[6] Ramezani M, Hosseinzadeh H, Daneshmand N (2001). Antinociceptive effect of Elaeagnus angustifolia fruit seeds in mice. Fitoterapia.

[7] Abbasian A, Naseri M, Masoud A (2004). Anti-inflammatory effects of Urtica pilulifera l seeds extracts in the rat. Iranian J. Pharm. Res.

[8] Hajhashemi V, Ghannadi A, Hajiloo M (2010). Analgesic and anti-inflammatory effects of Rosa damascena hydroalcoholic extract and its essential oil in animal models. Iranian J. Pharm. Res.

[9] Evans WC (2002). Trease and Evans Pharmacognosy.

[10] Zargari A (1990). Medicinal Plants.

[11] Saitta M, Giuffrida D, La Torre GL, Potortì AG, Dugo G (2009). Characterisation of alkylphenols in pistachio (Pistacia vera L) kernels. Food Chem.

[12] Giner-Larza EM, Máñez S, Recio MC, Giner RM, Prieto JM, Cerdá-Nicolás M, Ríos JL (2001). Oleanonic acid, a 3-oxotriterpene from Pistacia, inhibits leukotriene synthesis and has anti-inflammatory activity. Eur. J. Pharmacol.

[13] Orhan I, Aslan M, Sener B, Kaiser M, Tasdemir D (2006). In-vitro antiprotozoal activity of the lipophilic extracts of different parts of Turkish Pistacia vera L. Phytomedicine.

[14] Hosseinzadeh H, Mirshojaeian M, Razavi BM (2008). Antiemetic effect of Pistacia vera L. (Pistachio) leaves and nuts aqueous extracts in young chicken. Pharmacologyonline.

[15] Mansouri SMT, Naghizadeh B (2005). The effect of Pistacia vera L. gum extract on oxidative damage during experimental cerebral ischemia-reperfusion in rats. Iranian Biomed. J.

[16] Zhao X, Sun H, Hou A, Zhao Q, Wei T, Xin W (2005). Antioxidant properties of two gallotannins isolated from the leaves of Pistacia weinmannifolia. Biochim. Biophys. Acta.

[17] Wei T, Sun H, Zhao X, Hou J, Hou A, Zhao Q, Xin W (2002). Scavenging of reactive oxygen species and prevention of oxidative neuronal cell damage by a novel gallotannin, pistafolia A. Life Sci.

[18] Parvardeh SNM, Hosseinzadeh H (2002). Hepatoprotective activity of Pistacia Vera L gum extract in rats. J. Med. Plants.

[19] Parvardeh SNM, Nassiri AM, Hosseinzadeh H (2002). Antinociceptive, anti-inflammatory and acute toxicity effects of Pistacia Vera L. gum extract in mice and rats. J. Med. Plants.

[20] Monaco P, Previtera L, Mangoni L (1982). Terpenes in pistacia plants: A possible defence role for monoterpenes against gall-forming aphids. Phytochemistry.

[21] Ansari SH, Qadry JS, Ali M (1993). Essential oils of Pistacia integerrima galls and their effect on the central nervous system. Int. J. Pharmacog.

[22] Kawashty SA, Mosharrafa SA, El-Gibali M, Saleh NA (2000). The flavonoids of four Pistacia species in Egypt. Biochem. Syst. Ecol.

[23] Shi Q, Zuo C (1992). Chemical components of the leaves of Pistacia chinensis Bge. Zhongguo Zhong Yao Za Zhi.

[24] Kusmenoglu S, Baser KHC, Ozek T (1995). Constituents of the essential oil from the hulls of Pistacia vera L. J. Essent. Oil Res.

[25] Hou AJ, Peng LY, Liu YZ, Lin ZW, Sun HD (2000). Gallotannins and related polyphenols from Pistacia weinmannifolia. Planta Med.

[26] Orhan I, Kupeli E, Aslan M, Kartal M, Yesilada E (2006). Bioassay-guided evaluation of anti-inflammatory and antinociceptive activities of pistachio, Pistacia vera L. J. Ethnopharmacol.

[27] Hosseinzadeh H, Haddadkhodaparast MH, Arash AR (2003). Antinociceptive, antiinflammatory and acute toxicity effects of Salvia leriifolia Benth seed extract in mice and rats. Phytother. Res.

[28] Woolfe G, MacDonald AD (1944). The evaluation of the analgesic action of pethidine hydrochloride (demerol). J. Pharmacol. Exp. Ther.

[29] Cao BJ, Meng QY, Ji N (1992). Analgesic and anti-inflammatory effects of Ranunculus japonicus extract. Planta Med.

[30] Siegmund EA, Cadmus RA, Lu G (1957). Screening of analgesics including aspirin type compound based upon theantagonism of chemically induced writhing in mice. J. Pharmacol. Exp. Ther.

[31] Ansari SH, Ali M, Qadry JS (1994). New tetracyclic triterpenoids from Pistacia integerrima galls. Pharmazie.

[32] Duru ME, Cakir A, Kordali S, Zengin H, Harmandar M, Izumi S, Hirata T (2003). Chemical composition and antifungal properties of essential oils of three Pistacia species. Fitoterapia.

[33] Standen MD, Myers SP (2004). The roles of essential oils in the modulation of immune function and inflammation: Survey of aromatherapy educators. Int. J. Aromatherapy.

[34] Loomis T (1968). Essential of Toxicology.

[35] Parkhouse J, Pleuvry B J (1979). Analgesic Drug.

[36] Vogel HG, Vogel WH (1997). Drug Discovery and Evaluation, Pharmacological Assay.

